# Targeting CB2 and TRPV1: Computational Approaches for the Identification of Dual Modulators

**DOI:** 10.3389/fmolb.2022.841190

**Published:** 2022-02-25

**Authors:** Paula Morales, Chanté Muller, Nadine Jagerovic, Patricia H. Reggio

**Affiliations:** ^1^ Medicinal Chemistry Institute, Spanish National Research Council, Madrid, Spain; ^2^ Department of Chemistry and Biochemistry, University of North Carolina at Greensboro, Greensboro, NC, United States

**Keywords:** cannabinoids, ionotropic receptors, CB2, TRPV1, dual ligands, multitargeting

## Abstract

Both metabotropic (CBRs) and ionotropic cannabinoid receptors (ICRs) have implications in a range of neurological disorders. The metabotropic canonical CBRs CB1 and CB2 are highly implicated in these pathological events. However, selective targeting at CB2 versus CB1 offers optimized pharmacology due to the absence of psychoactive outcomes. The ICR transient receptor potential vanilloid type 1 (TRPV1) has also been reported to play a role in CNS disorders. Thus, activation of both targets, CB2 and TRPV1, offers a promising polypharmacological strategy for the treatment of neurological events including analgesia and neuroprotection. This brief research report aims to identify chemotypes with a potential dual CB2/TRPV1 profile. For this purpose, we have rationalized key structural features for activation and performed virtual screening at both targets using curated chemical libraries.

## 1 Introduction

Well documented pharmacological evidence supports functional crosstalk between the endocannabinoid system (ECS) and the endovanilloid system (EVS) (Di Marzo et al., 2002; Lastres-Becker et al., 2003; Morgese et al., 2007; Avraham et al., 2010; Chávez et al., 2010; Adamczyk et al., 2012; Arnold et al., 2012; Lowin and Straub, 2015; Rossi et al., 2015; Malek and Starowicz, 2016; Assimakopoulou et al., 2017; Bellini et al., 2017; Zhang et al., 2017; Punzo et al., 2018; Bhatta et al., 2019; Wi et al., 2020). Thus, these latest advances provide opportunities to develop innovative strategies for fighting disorders where biological targets of both systems are involved. Here, we emphasize the cannabinoid receptor type 2 (CB2) and the transient receptor potential vanilloid type 1 (TRPV1) channel, both implicated in neurodegenerative diseases and pain.

CB2R is a G-protein-coupled receptor (GPCR) mainly present in the immune cells where they are expressed in lymphocytes, natural killer cells, macrophages, and neutrophils (Cécyre et al., 2020). Thus, they are an attractive target for the treatment of inflammatory processes. The expression of CB2 is also detected in the central nervous system (CNS) under stressful conditions such as cytotoxic and neuroinflammatory injuries within the brainstem, microglia, and astrocytes, suggesting CB2 an interesting target for neuroprotection (Navarro et al., 2016). CB2 is also expressed in the blood brain barrier (BBB), and therefore could be beneficial in the brain and peripheral tissues at different stages of neurodegenerative processes (Aso and Ferrer, 2016; Javed et al., 2016; Cassano et al., 2017; Behl et al., 2020; Berry et al., 2020; Uddin et al., 2020). CB2 selective agonists also represent an attractive approach for pain management among other therapeutic applications (Fowler, 2020). In animal models of chronic inflammation, CB2 agonists lead to beneficial outcomes for diverse pain managements such as neuropathic, osteoarthritic, postoperative, and human immunodeficiency virus (HIV) associated pain relief (Guindon and Hohmann, 2008; Anthony et al., 2020; Aly and Masocha, 2021; Bryk and Starowicz, 2021; Mlost et al., 2021; Ramírez-López et al., 2021).

TRPV1 is a nonselective cation channel mainly expressed in the sensory neurons of the peripheral nervous system (Caterina et al., 1997), acting as a detector of painful stimuli such as heat and pungent chemicals like capsaicin. TRPV1 modulators have attracted much attention as analgesics due to its implication in pathological pain such as inflammatory, visceral, neuropathic, and cancer-related pain (Peppin and Pappagallo, 2014; Malek and Starowicz, 2016; Shuba, 2021). TRPV1 has also been described in the CNS (Gibson et al., 2008; Ho et al., 2012; Shuba, 2021) with expression in neurons, microglia, and astrocytes (Sawamura et al., 2017), and its level of expression can be up- or down-regulated according to age and pathophysiological conditions (Martins et al., 2014). TRPV1 participates to the regulation of neuronal function and synaptic plasticity (Marinelli et al., 2003; Maione et al., 2009; Chávez et al., 2010), the control of motor behavior (Morgese et al., 2007; González-Aparicio and Moratalla, 2014; Martins et al., 2014), and the regulation of neuroinflammation (Kong et al., 2017). Therefore, TRPV1 has been suggested to be implicated in diseases associated with motor dysfunctions, such as Huntington’s, Parkinson’s, and multiple sclerosis, or with cognitive functions like Alzheimer’s disease (González-Aparicio and Moratalla, 2014; Nam et al., 2015; Li et al., 2019; Du et al., 2020).

Co-expression and crosstalk between TRPV1 and CB1 (Cristino et al., 2006; Assimakopoulou et al., 2017) has been established primarily in the modulation of arthritic pain and inflammation (Lowin and Straub, 2015). In addition to CB1, CB2 is also co-expressed with TRPV1 in certain cells including osteoblasts (Rossi et al., 2015), osteoclasts (Bellini et al., 2017), and sensory neurons (Wi et al., 2020). Moreover, CB2 and TRPV1 crosstalk has shown to be engaged diverse pathophysiological processes including pain (Wi et al., 2020; Wilkerson et al., 2022), bone disorders (Rossi et al., 2015; Bellini et al., 2017), inflammatory processes (Lowin et al., 2016; Arnold et al., 2021), cocaine-seeking behavior (Adamczyk et al., 2012), proliferation and apoptosis of T-lymphoblastic leukemia cells (Punzo et al., 2018), and multidrug resistance (Arnold et al., 2012). Benefits of the CBR/TRPV1 axis for neurodegenerative diseases has been suggested by some studies due to CBRs and TRPV1 inhibition of glial activation and expression of proinflammatory cytokines in a mouse model of Parkinson’s disease (Wi et al., 2020). Pharmacologically, strategies targeting CB1/TRPV1 have shown promising therapeutic results in models of pain, spasticity, arthritis, and dyskinesia (Di Marzo et al., 2001, 2002; Brooks et al., 2002; Morgese et al., 2007; Lowin and Straub, 2015) For instance, arvanil, a CB1 agonist, TRPV1 activator, and potent inhibitor of anandamide (AEA) accumulation, alleviates hyperkinesia typical of Huntington’s disease (De Lago et al., 2005). However, few reports have identified dual CB2/TRPV1 modulators thus far.

Current treatments for complex disorders based on selective-target drugs fail in their efficacy. As a consequence, a number of research studies have highlighted the importance of multiple-target strategies for the treatment of multifactorial disorders such as pain and neurodegenerative diseases (Cheong et al., 2019; Gontijo et al., 2019; Maramai et al., 2020). Combinatorial therapies are generally associated with side effects derived from drug-drug interactions. Therefore, single dual-acting drugs should reduce side effects with unique pharmacokinetic or pharmacodynamic profiles. Cannabinoids have been reported to directly modulate TRPV1 (Muller et al., 2019), and among them, few have shown selective CB2 vs CB1 activity. In this brief research report, we will primarily focus on the *in silico* identification of potential CB2/TRPV1 chemotypes, as well as rationalize reported dual modulators.

## 2 Methods and Materials

### 2.1 Receptor Structures

Structures of *h*CB2 and *h*TRPV1 were selected based on the reliability and stability of the structures. In a recent publication, an activated structure of *h*CB2 was resolved via cryo-EM at a resolution of 2.90 Å (PDB: 6KPF) (Hua et al., 2020). This structure was used for our docking screening upon treatment using the protein structure preparation wizard integrated in the Schrödinger software. A model of *h*TRPV1 was constructed using the cryo-EM structure PDB: 5IRZ congruent to the methods described in Muller et al. and was used for this work (Muller et al., 2020).

### 2.2 Grid Generation

Prior to using the Glide module high-throughput virtual screening (HTVS) and extra precise (XP) docking within the Schrödinger package (Schrödinger, LLC, New York, NY, 2019), docking grids were generated using the receptor grid generation tool within Glide to ensure ligand screening was performed in the appropriate sites within each receptor. Dimensions for the CB2 receptor grid were set at 20 Å in length along the *x*, *y*, and *z* axes and was centered on the ligand co-crystallized with the CB2 structure (the THC synthetic derivative AM12033).

Similarly, all three TRPV1 grids were generated to adhere to the same dimensions of 20 Å in length in the *x*, *y*, and *z* directions and were centered on residues that are believed and/or reported to be involved with ligand binding at each location. This resulted in three distinctly different grids for TRPV1 that will herein be referred to as “VBP” for the location that capsaicin binds, “tunnel” for the location where anandamide has been reported to interact with TRPV1 via MD simulations, and “CBD-site” for the putative CBD interaction site reported in the TRPV2/CBD cryo-EM structure. Visual representations and further explanation of these TRPV1 sites can be found in Supplementary Figure S1. These grid specifications allow any ligand that is less than or equal to 20 Å in length to be docked within the specified region.

### 2.3 Curation of Chemical Libraries

#### 2.3.1 CB2

From the CB2 indexed molecules, ligands showing EC_50_, Emax, and activity data were selected (total of 6356) and retrieved from the ChEMBL webserver as a .csv file. The “activity” category includes compounds with not only agonist activity, but antagonist, inverse agonists, and allosteric modulators as well. DataWarrior, an open-source data visualization software, was used to further analyze the ligand output which included discarding ligands without an agonist profile (−568 ligands), removing duplicates (−2159 ligands), and eliminating ligands with low activity (−773 ligands). This resulted in a final CB2 library of 2856 unique molecules that included a variety of chemotypes.

#### 2.3.2 TRPV1

Ligands that have been indexed for TRPV1 activity within the ChEMBL database were selected and filtered in search of agonists in accordance with the reported EC_50_ and E_max_ values and activity. The resulting 7,436 compounds were exported from the ChEMBL webserver as a .csv file and uploaded to DataWarrior. The selection of ligands with TRPV1 activity from the ChEMBL database included antagonists, inverse agonists, possible allosteric modulators, ligands with low activity, and duplicates which were all removed using DataWarrior. The final curated TRPV1 library contained 3,830 unique molecules with a variety of chemotypes.

#### 2.3.3 Internal Standard Ligands

The CB2 agonist resolved with the active *h*CB2 structure (AM12033) was used as an internal standard for CB2 docking. Three internal standards were used for hTRPV1: capsaicin in the VBP, AEA in the tunnel as observed from MD simulations, and CBD at the putative CBD site.

#### 2.3.4 JWH133 Similarity Library

JWH133, which acts as an agonist at both CB2 and TRPV1, was used as a molecular basis for this additional screen to explore more unique scaffold options that may not be present in the CB2 or TRPV1 curated libraries. A JWH133 similarity library was curated using PubChem Biosays, 2021 which included compounds that shared >0.85 Tanimoto similarity index with JWH133, while also following Lipinsky’s rules of drug likeness (apart from xLogP values, which were set to −1 to 6 due to the lipophilicity of cannabinoid ligands). The JWH133 similarity library consisted of 5081 that were screened at all sites (CB2 and the three TRPV1 sites), and the output was analyzed to identify dual potential chemotypes.

### 2.4 High-Throughput Virtual Screening Workflow

A general overview of the screening workflows is provided in Supplementary Figure S2.

#### 2.4.1 Ligand Preparation

Each of the curated libraries were exported as .sdf files and their conformations were optimized using the LigPrep module of the Maestro suite (Schrödinger, LLC, New York, NY, 2019). The Epik software was employed to predict pKa values in the pH range of 7.0 ± 0.5 and to return all chemically sensible structures in accordance with the Hammett and Taft methodology. All compounds were minimized using the OPLS3e force field as implemented in Maestro.

#### 2.4.2 HTVS

Molecular docking was performed using the HTVS Glide-dock module integrated in the Schrödinger package. The HTVS was conducted under the default setting, ensuring that high-energy ionization and tautomer states were removed, and the planarity of conjugated pi systems were enhanced. Ligands were docked flexibly, allowing for exploration of an arbitrary number of torsional degrees of freedom, in addition to the six spatial degrees of freedom spanned by the translational and rotational parameters. Up to 10 poses per compound state were generated and ligand poses that were generated in this way were run through a series of hierarchical filters to evaluate ligand interactions with the receptor. Docking score, glide gscore, glide emodel, ionization penalty, and topological polar surface area (TPSA) were used to select the docking poses in the output. The output from the HTVS contained the top 10% of the best scoring compound states and were analyzed for use in the extra precise (XP) screen via their docking scores.

#### 2.4.3 XP Screening

Top scoring compounds from the HTVS were then studied through high-precision docking calculations which was performed using the XP Glide module. As with the HTVS protocol, 10 poses of the short-listed ligands were docked flexibly in their respective receptor site within the generated grids. A post-docking minimization was performed and the top 20% of the best scoring ligands were retained. XP Glide uses two key features that impact the XP Glide scoring: the recognition of structural motifs that provide large contributions to binding affinity and the application of large desolvation penalties to ligand and protein polar and charged groups wherever appropriate. To accomplish this, the sampling algorithm and scoring functions have been simultaneously optimized in XP. Ligands making it through the XP screen were organized by their docking scores and analyzed for ligand/receptor interactions. Selected ligands for each receptor were investigated through manual docking based on the automatic docking score, binding mode, as well as reported activity.

#### 2.4.4 Additional Criteria

*Manual Docking Identification of Potential PAINS Off-Targets Evaluation*. Selected compounds were subjected to manual docking at CB2 and TRPV1 for further investigation of key interactions. Docking at CB2 was performed following the protocols previously reported by us for cannabinoid and related GPCRs (Morales et al., 2017a). In the case of TRPV1, select ligands were positioned within the respective binding site with steric clashes being removed via ligand and/or receptor adjustment using a graphical interface. Minimization of the ligand and surrounding 6 Å of residues (due to complex size) was performed using Prime version 19.3 (Schrödinger Inc.) with the OPLS3e forcefield in an implicit membrane.

*In silico calculation of ADME properties.* A set of 34 physico-chemical descriptors was computed using QikProp version 3.5 integrated in Maestro (Schrödinger, LLC, New York, United States). The QikProp descriptors are shown in Supplementary Tables S2, S3. The 3D conformations used in the calculation of QikProp descriptors were generated using LigPrep as previously detailed.

*Identification of Potential PAINS*. In the search of potential candidates, it is crucial to avoid the presence of potential promiscuous moieties or PAINS (pan-assay interference compounds) (Baell and Holloway, 2010; Capuzzi et al., 2017). Therefore, the selected molecules were subjected to a PAINS identification study using the swissADME webserver (Daina et al., 2017).

*Off-Targets evaluation*. XP Glide docks at potential off-target receptors including cannabinoid-related GPCRs such as CB1 (Shao et al., 2016; Hua et al., 2020), GPR55 (Kotsikorou et al., 2013; Lingerfelt et al., 2017), GPR18 (Sotudeh et al., 2019) and TRP channels such as TRPV2 (Pumroy et al., 2019), TRPV3 (Singh et al., 2018; Zubcevic et al., 2018), TRPA1 (Suo et al., 2020; Zhao et al., 2020), and TRPM8 (Diver et al., 2019; Yin et al., 2019). For this purpose, the cited available structures, whether crystal, cryoEMs, or models previously developed in our group, have been used. Results of these additional dockings can be found in Supplementary Tables S4, S5.

## 3 Results and Discussion

Polypharmacological approaches targeting the ECS have already shown successful results in diverse disease models (Fernández-Fernández et al., 2014; Malek and Starowicz, 2016; Barutta et al., 2017; Lago-Fernandez et al., 2021). However, drug discovery strategies primarily targeting CB2 and TRPV1 have not yet been explored. As previously detailed, activation of these targets participates in diverse therapeutic effects including analgesia and neuroprotection, which both offer interesting polypharmacological prospects.

### 3.1 Structural Understanding of Compounds With Reported Activity at Both Targets

To computationally identify promising chemotypes with a CB2/TRPV1 dual agonist profile we have first analyzed reported compounds exhibiting activity at both receptors. As detailed in Supplementary Table S1, endocannabinoids, phytocannabinoids, and their respective synthetic derivatives have so far shown the best promise in this field.

The well-known endogenous ligands 2-arachidonoylglycerol (2-AG) and anandamide (AEA) exhibit agonist effects at both targets with low micromolar potency. As observed in diverse *in vitro* and *in vivo* models, these endocannabinoids also display activity at other cannabinoid-related GPCRs including CB1, GPR55, and GPR18 (Morales and Jagerovic, 2016; Morales and Reggio, 2017; Morales et al., 2020) as well as other TRP channels including TRPA1 and TRPM8 (Muller et al., 2019).

Synthetic endocannabinoid-like derivatives have also shown dual activity (Supplementary Table S1). For instance, Appendino and coworkers reported a series of conformationally constrained fatty-acid ethanolamides with CB1, CB2, and TRPV1 activity (Appendino et al., 2009). An example from this series is ACPA-OH (Supplementary Table S1), which introduces a hydroxycyclopropyl in the amide head group forcing a specified stereochemistry and rigidity. This compound is a potent TRPV1 agonist that exerts low micromolar CB2 affinity and nanomolar binding at CB1 (Appendino et al., 2009). Further synthetic efforts from Di Marzo’s research group led to the identification of hybrid cannabinoid-vanilloid ligands with a highly CB1 selective profile (Melck et al., 1999; Szallasi and Di Marzo, 2000; Di Marzo et al., 2001, Di Marzo et al., 2002). Among these fatty-acid derivatives, one of the few compounds that binds to CB2 is O-1811 (Supplementary Table S1), which presents a substituted dimethyl-hydroxyhexanyl tail (Di Marzo et al., 2001). Despite targeting CB2, O-1811 displays over 6-fold CB1 selectivity.

Interestingly, molecules combining the polyunsaturated fatty-acid chain with the vanillyl-amide head group of capsaicin behave as CB1/TRPV1 agonists that potently inhibit anandamide accumulation (Melck et al., 1999; Szallasi and Di Marzo, 2000; Di Marzo et al., 2001, 2002). One such molecule, arvanil (Supplementary Table S1), has shown therapeutic potential in the treatment of dyskinesia associated to Huntington’s disease (De Lago et al., 2005) and inhibition of spasticity and persistent pain (Brooks et al., 2002).

Structural modifications in the long chain of endocannabinoid-like molecules led to the identification of the first series of CB2 selective/TRPV1 dual ligands (Appendino et al., 2006). Combination of non-polyunsaturated fatty acid-derived chains with 12-acylgroups yielded compounds such as 12-phenylacetylricinoleyl cyclopropylamide (PhAR derivative 12, Supplementary Table S1) which behaves as a potent TRPV1 agonist and CB2 inverse agonist.

Diverse phytocannabinoids have also shown activity at CB2 and TRPV1 (De Petrocellis et al., 2011; Zagzoog et al., 2020). For instance, the main non-psychotropic component of *Cannabis sativa*, cannabidiol (CBD), is a CB2 partial agonist/TRPV1 agonist (De Petrocellis et al., 2011; Tham et al., 2018). It is worth mentioning that at CB2, CBD has been reported to act as negative allosteric modulator in the presence of orthosteric full agonists (Martínez-Pinilla et al., 2017; Navarro et al., 2021). The acidic CBD derivative, cannabidiolic acid (CBDA), and its propyl counterpart, cannabivarin (CBDV), also exhibited TRPV1 agonism while being CB2 partial agonists (De Petrocellis et al., 2011; Zagzoog et al., 2020). The phytogenic compound cannnabigerol (CBG) presents the same functional profile at both targets (De Petrocellis et al., 2011; Zagzoog et al., 2020). On the other hand, the well-known psychoactive compound tetrahydrocannabinol (THC) is not active at TRPV1 (De Petrocellis et al., 2011), whereas its propyl derivative tetrahydrocannabivarin (THCV) behaves as a TRPV1/CB2 agonist (Supplementary Table S1). It is important to note that all these phytocannabinoids also display activity at CB1 receptors.

Synthetic phytocannabinoid-like derivatives have also shown interesting dual activity. The CB2 selective agonists HU308 and JWH133 could be considered dual ligands due to their activity at TRPV1 being HU308 a weaker agonist at this channel (Soethoudt et al., 2017). The widely used aminoalkylindole WIN55212-2, which is a potent CB1/CB2 synthetic agonist, has also been reported to activate and desensitize TRPV1 (Soethoudt et al., 2017).

In the search of novel structures with CB2/TRPV1 activity, we aim to minimize off-target effects at CB1 or related receptors. Therefore, considering the aforementioned reported activity, we selected JWH133 as a molecular basis for the identification of potential dual CB2/TRPV1 agonists. The therapeutic potential of this ligand has been recently reviewed elsewhere (Agonist et al., 2021). As a first step we rationalized its interactions at both receptors using molecular docking. At CB2 JWH133 sits in the orthosteric pocket with the same orientation as the CB2 agonist resolved in the cryoEM structure AM12033 (Supplementary Figure S3A). The tricycle stablishes π-π stacking with residues F2.61, F2.57 and F183 (extracellular loop 2) while residues I3.29, F2.64 and V3.32 stabilize the molecule through van der Waals interactions. The orientation of the distal aliphatic tail of JWH133 differs from that of AM12033 due to the lack of a functional group at the end. As in the case of AM12033 the so-called twin toggle switch residues F3.36 and W6.48 (Hua et al., 2020) are stabilized in their active conformation as shown in Supplementary Figure S3A. In TRPV1, JWH133 cleared both HTVS and XP screening in what is thought to be the CBD binding site. CBD has yet to be co-resolved with TRPV1, though it has with TRPV2 and an analysis of the putative CBD binding site was performed across all ICRs (Muller et al., 2020). The CBD structure in TRPV2 displays a different orientation than the CBD screen at TRPV1, though the differences cited above could be responsible. CBD and JWH133 show similar π-π stacking with Y584, though CBD is also stabilized by F639, likely due to the central constraint of JWH133 which angles the ligand slightly outward (Supplementary Figure S3B).

### 3.2 Towards the Identification of Potential Dual Ligands

In order to identify potential chemotypes with a yet unexplored TRPV1/CB2 dual profile two different *in silico* approaches have been followed. These strategies are described in the following subsections and the workflows are depicted in Supplementary Figure S2.

#### 3.2.1 Virtual Screening of JWH133 Structurally Related Chemical Databases

A chemical library of compounds with >0.85 Tanimoto similarity index with JWH133 was curated and screened at CB2 and TRPV1 using the methods described above (workflow depicted in Supplementary Figure S2A). Analysis of docking interactions of top-ranked XP results from the CB2 site and the TRPV1 sites revealed seventeen common ligands between the CB2 site and the VBP and CBD sites (Table 1). No common ligands were identified between the CB2 site and the TRPV1 tunnel. The selection of novel potential CB2/TRPV1 chemotypes includes key structural features and ligand-receptor site interactions at both targets, as well as the absence of previously reported activity at these receptors. This strategy allows for prioritization of molecularly diverse and novel compounds. To ensure the VBP and CBD sites were explored equally, two ligands were selected that targeted CB2 and the VBP (57756957 and 151332252), two ligands were selected that targeted CB2 and the CBD site (59824268 and 123533625), and one ligand was selected that targeted CB2, the VBP, and the CBD site (153641693), resulting in the selection of five ligands for further investigation via manual docking and pharmacokinetic profiling (Supplementary Table S2). The selected chemotypes have not been yet explored at CB2/TRPV1 and their reported activity is not significant, providing novel opportunities for the investigation of the endocannabinoid system.

**TABLE 1:** Potential dual CB2/TRPV1 candidates obtained upon screening of a JWH133 structurally related chemical database. Selected hits have been classified according to common structural moieties.

PubChem ID	Structure	CB2 docking score	TRPV1 docking score		Reported biological activity	References
			VBP site	CBD site		
JWH133	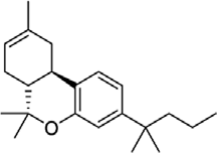	−10.24	−7.05	−6.54	CB2/TRPV1 reference agonist	(Huffman et al., 1999; Soethoudt et al., 2017)
*4-Aryl chromanes*						
1238803	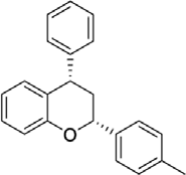	−10.35	−8.21	-	Synthetic methodology, no activity reported^a^	Liu et al. (2016)
6577075	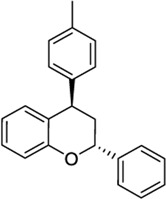	−10.49	−8.04	-	Synthetic methodology, no activity reported^a^	Liu et al. (2016)
7066525	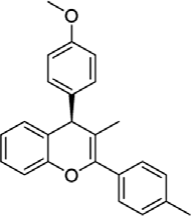	−10.13	-	−8.25	No activity reported^a^	Commercially available
20560217	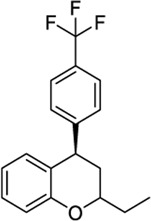	−10.03	−8.79	-	Anorexigenic activity in rats^a^	Sime and Ainsworth, (1981)
57756957^b^	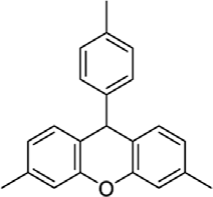	−10.23	−8.73	-	Bactericide and antiviral activity^a^	Habi et al. (2001)
*3- or 7-Methylene chromanes*						
59824268^b^	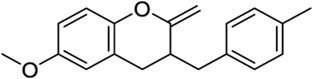	−9.78	-	−8.22	Ink composition^a^	Kazuhiro, (2008)
148365500	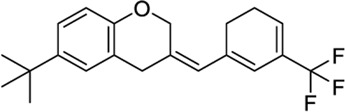	−10.44	-	−8.81	Electrolyte composite for a fuel cell containing a fluorine ion-exchange resin^a^	Kim et al. (2011)
91587558	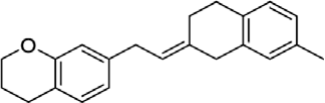	−10.24	−8.18	-	Modulator of dopamine 3 receptor^a^	Wang et al. (2010)
141098199	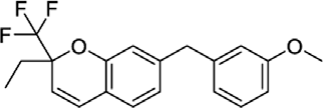	−10.28	−8.72	-	Anti-inflammatory properties^a^	Carter et al. (2004)
*Phytocannabinoid-like molecules*						
68117155	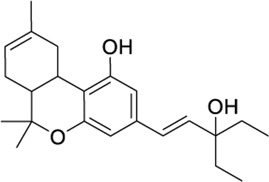	−10.88	−8.99	-	Phytocannabinoid-like molecule claimed as tranquilizing and antidepressant agent	Razdan and Dalzell, (1976)
142557024	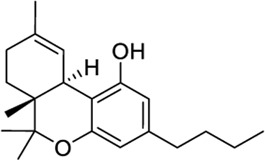	−10.30	−8.52	-	Phytocannabinoid-like molecule included in a cannabinoid preparation that contains α-tocopherol	Karsten et al. (2019)
148053384	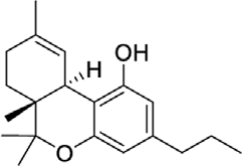	−10.29	−8.29	-	Topical compositions comprising hydroxy acids and cannabinoids for skincare	Ghalili and Mcgovern (2016), Viet Thang Vu et al. (2019), Yong-Gang et al. (2019)
*Other tricyclic structures*						
89342940	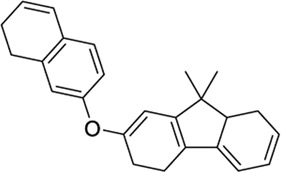	−10.69	-	−8.53	Organic luminescent material^a^	Funahashi et al. (2007)
123533625^b^	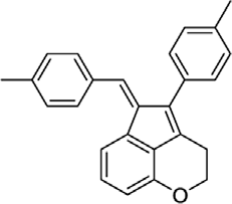	−10.81	-	−9.50	Intermediate in the modular synthesis of graphene nanoribbons^a^	Byers and Alabugin, (2012), Alabugin and Byers (2015)
153641693^b^	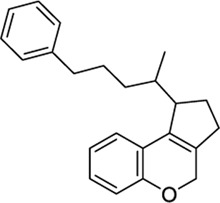	−10.12	−8.49	−8.74	Synthesis of heterocyclic esters of benzopyrans, no activity reported^a^	Winn (1974)
*Miscellaneous chemotypes*						
140022260	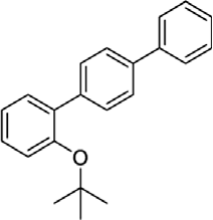	−10.16	−8.75	-	Synthesis of new 4,4”-substituted oxy-p-terphenyl compounds, no activity reported^a^	Koji and Hiroyasu, (2005)
151332252^b^	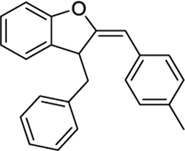	−10.35	−9.79	-	Synthesis of 2-substituted 3-arylmethylbenzofuran, no activity reported	Ying-Ji and Bo-Yuan, (2013)

a CB2 and TRPV1 activity has not been reported for these compounds. Docking scores are provided in Kcal/mol.

b Molecules selected for further investigations through manual docking and ADMET profiling.

**FIGURE 1:**
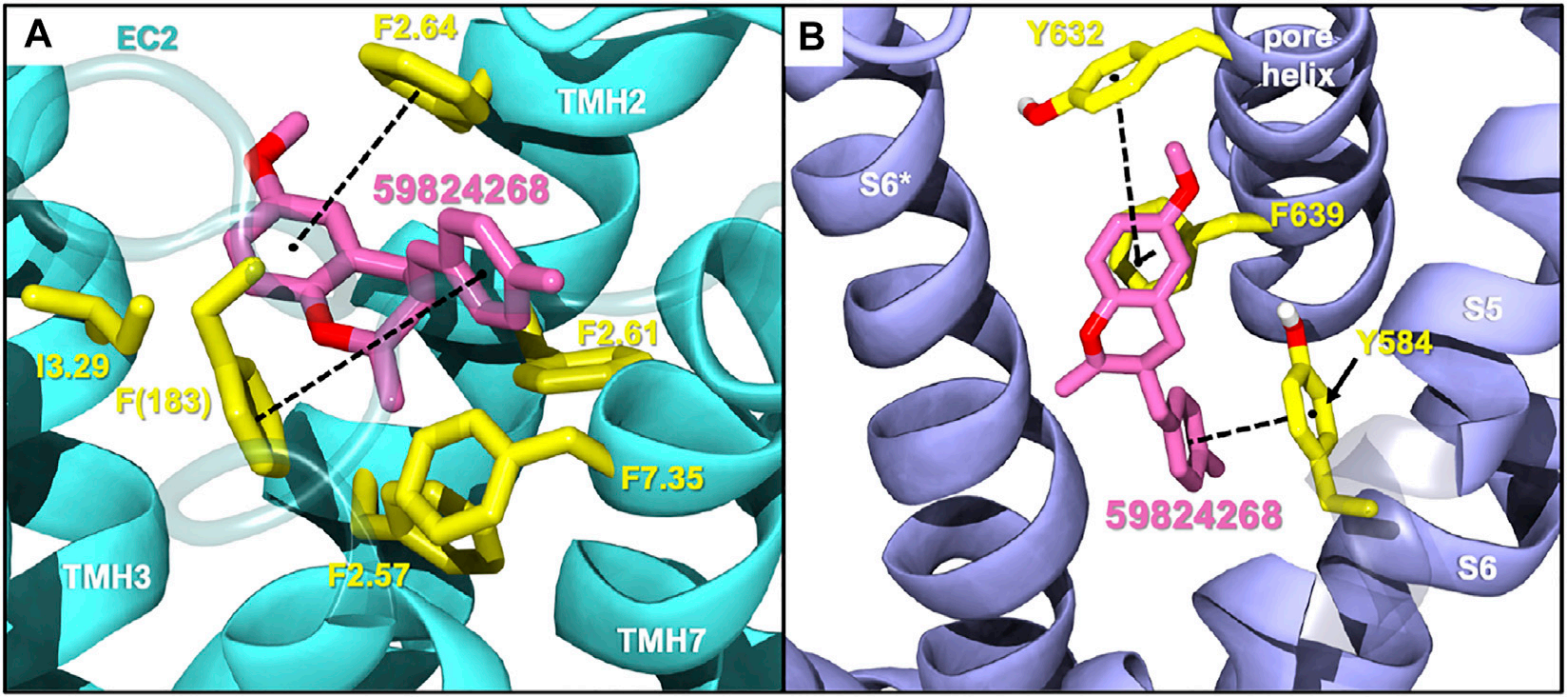
Selected compound 59824268 docked in CB2 **(A)** and TRPV1 **(B)**. EC2: Extracellular loop 2; TMH, transmembrane helix.

Among the five selected candidates, 3-(4-methylbenzyl)-chromane 59824268 presented a better druggability profile (Supplementary Table S2) being therefore prioritized for future *in vitro* testing as CB2/TRPV1 dual modulator. As shown in Supplementary Table S2, candidates 57756957, 123533625, 153641693, and 151332252 exhibit HERG values that fall outside the range of approved drugs. Docking studies of 59824268 at CB2 and TRPV1 are shown in Figure 1. At CB2 this chromane sits a bit higher than JWH133 in the binding crevice being stabilized by hydrophobic and aromatic interactions with residues F2.64, F2.61, F2.57, F183 and F7.35. Regarding TRPV1, 59824268 orients itself in a way similar to JWH133 in the pocket maintaining overlap with the aromatic ring. While JWH133 appears to have primary interactions with Y584, 59824268 has interactions with Y584 in addition to Y632 and F639, further stabilizing the chromane in this pocket.

#### 3.2.2 Cross-Agonist Virtual Screening

The second strategy for the identification of dual compounds is based on a HTVS of reported CB2 and TRPV1 agonists. CB2 agonists indexed in the ChEMBL database have been retrieved and studied in the three known TRPV1 binding sites as detailed in section 2.4. Likewise, TRPV1 ligands indexed in the ChEMBL database have been retrieved and studied in the CB2 binding site. Following the workflow depicted in Supplementary Figure S2B, five candidates were selected for further analysis at each receptor (Table 2). Reported activity at the known target, docking score (Table 2) and druggability profile (Supplementary Table S3) led us to select compounds 1288208, 1288239 (TRPV1 virtual screening) 1508577 and 1508215 (CB2 virtual screening).

**TABLE 2:** Potential dual CB2/TRPV1 candidates obtained through the crossed-agonist strategy.

ChEMBL ID	Structure	CB2	TRPV1 reported activity	Other reported targets	References
		Docking score			
AM12033	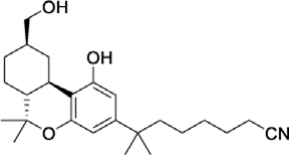	−12.61	NR	None reported	Hua et al. (2020)
1508577	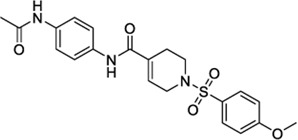	−11.81	EC_50_ = 648.4 nM	Inhibitor of the malarial parasite plastid	(PubChem Bioassays)^a^
1508215	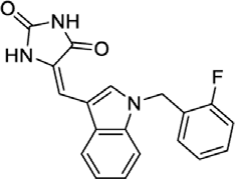	−11.67	EC_50_ = 23.0 nM	Aldehyde Dehydrogenase 1	(PubChem Bioassays)^a^
1574712	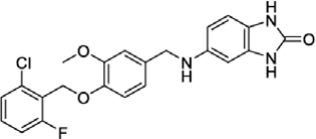	−11.24	EC_50_ = 2581.2 nM	None reported	(PubChem Bioassays)^a^
1383349	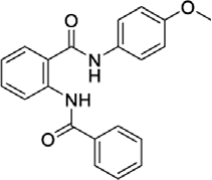	−11.18	EC_50_ = 81623.2 nM	None reported	(PubChem Bioassays)^a^
1347563	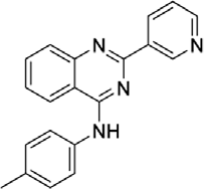	−10.89	EC_50_ = 1451.5 nM	Inhibitors of the malarial parasite plastid, tyrosyl-DNA phosphodiesterase 1 and TGF-β	(PubChem Bioassays)^a^
**ChEMBL ID**	**Structure**	**CB2**	**TRPV1**	**Other reported targets**	**References**
		**Reported activity**	**Docking score + site**		
AEA	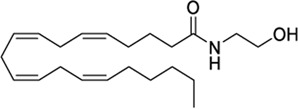	EC_50_ = 0.43 μM	−5.01 tunnel	CB1, PPARs, FAAH	McPartland et al., (2007), Soethoudt et al. (2017)
		K_i_ = 0.44 μM^b^			
1288208^c^	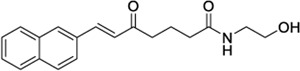	K_i_ =1.03 μΜ	−8.55 tunnel	No activity at CB1	Osman et al. (2010)
				No other target reported	
1288239	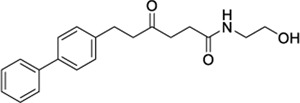	K_i_ =2.25 μM	−8.32 tunnel	No activity at CB1	Osman et al. (2010)
				No other target reported	
CBD	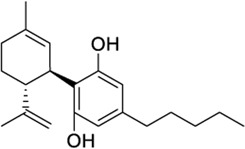	EC_50_ = 0.05 μM	−10.79 CBD	Several off targets	Morales et al. (2017b), Zagzoog et al. (2020), Navarro et al. (2021)
		K_i_ = 0.02–0.56 μM^b^			
1644371	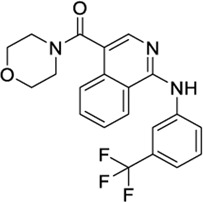	EC_50_ = 15.8 nM	−9.45 CBD	Weak CB1 activity	Saari et al. (2011)
3114522	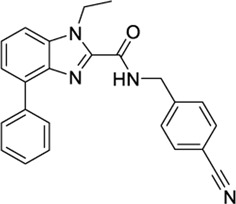	EC_50_ = 84 nM	−9.56 CBD	No activity at CB1	Nanda et al. (2014)
				No other target reported	
RTX	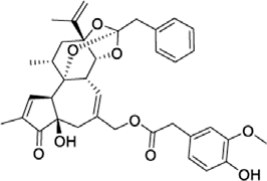	NR	−11.66 VBP	Analgesic	Brown (2016), Gao et al. (2016)
3353818	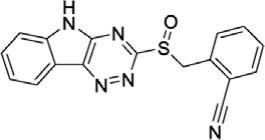	EC_50_ = 3.5 μM	−9.80 VBP	None reported	Gianella-Borradori et al. (2015)
1288208^c^	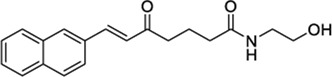	K_i_ = 1.03 μΜ	−9.97 VBP	No activity at CB1	Osman et al. (2010)
				No other target reported	

a PubChem bioassays: qHTS assay for compounds that act as agonists of TRPV1: hit validation.

b See Supplementary Table S1 for further pharmacological information.

c Compound selected for both tunnel and VBP docking.

NR: not reported

**FIGURE 2:**
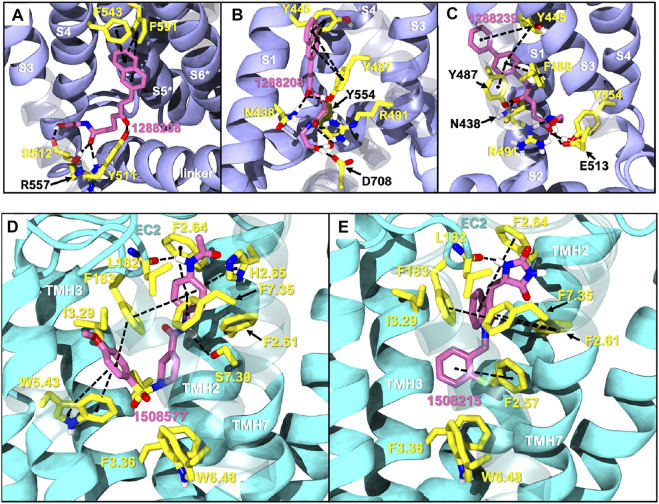
Docks of selected potential dual candidates: TRPV1 in purple cartoon ribbons **(A–C)** and CB2 in cyan cartoon ribbons **(D,E)**. Molecules are displayed in pink tubes; all interactions are shown via dashed lines and each helix and residue is labeled. **(A)** shows 1288208 in the VBP. A portion of S3 is transparent to aid in visibility; **(B)** shows 1288208 in the tunnel. Helix S2 is shown completely transparent to aid in the visibility of the tunnel; **(C)** shows 1288239 in the tunnel with a portion of helix S2 transparent to aid in visibility. **(D)** shows a lipid view of the 1508577/CB2 complex; **(E)** shows a lipid view of the 1508215/CB2 complex; TMH6 and 7 are displayed with transparency for a clearer view of the binding site.

1288208 passed the screening as a potential modulator of TRPV1 at two sites: the VBP and tunnel. While there is argument for the elimination of this ligand due to lack of site specificity, it was selected for exactly this reason. With the abundance of ligands reported to modulate TRPV1, and the variability in reported and putative binding locations, a ligand that shows the potential for interaction at multiple locations, both putative and confirmed, within the channel is worthy of further study to better understand why this is. In the VBP, the headgroup of 1288208 forms H-bonds with R557 and S512 via the backbone and hydroxy group, both reachable from within the tunnel. The α,β-unsaturated ketone oxygen H-bonds with Y511, and the addition of the naphthyl moiety at the tail end of the ligand provides pi-stacking capabilities farther up in the VBP with F543 and F591 (Figure 2A).

AEA docking in the tunnel shows headgroup interactions with several residues including Y554, Y555, Y487, D708, and N438 (Supplementary Figure S4). Like AEA, the hydroxyl portion of the headgroup of 1288208 maintains interactions with Y554 and D708, while additional H-bonding between R491 and the amide oxygen is present. The inclusion of an α,β-unsaturated ketone mid-tail allows for more H-bonding via Y487 and N438 near the entrance of the tunnel. The naphthyl moiety at the end of the tail displays pi-pi interactions with both Y487 and Y445 (Figure 2B). The additional interactions of 1288208 could aid in the stability of the ligand in the tunnel from an external standpoint, allowing the headgroup more time in the tunnel, potentially triggering channel activation as previously hypothesized from MD simulations (Muller et al., 2020, 2021).

The other selected TRPV1 ligand, 1288239 (Figure 2C), shows headgroup interactions with R491 and Y554, like 1288208, with an additional interaction with E513. The ketone found midway down the tail of ligand H-bonds with N438 and Y487, again similar to 1288208. One feature that differentiates 1288239 from 1288208 is a biphenyl moiety in place of a naphthyl moiety. The lower ring of the biphenyl moiety has aromatic interactions with Y487 and F488, and both rings interact with Y445.

Because of their high potency at TRPV1, their interaction pattern at the CB2 orthosteric pocket and their optimal drug-like properties, compounds 1508577 and 1508215 were selected as potential candidates in the *in silico* search of dual ligands. Compounds like ACPA-OH and JWH133 also ranked at the top, however, since we are looking for unexplored dual chemotypes, they were not selected in this *in silico* study. Consistent with the hydrophobic nature of the CB2 orthosteric pocket, compound 1508577 is mainly stabilized by aromatic and van der Waals interactions. As displayed in Figure 2D, π-π stackings are stablished between the methoxybenzene group with W5.43 and F183 and the phenylacetamide group with F183, F7.35, F2.64 and H2.65. Moreover, the acetamide hydrogen engages with the backbone carbonyl oxygen of V182 in a H-bond while the central amide H-bonds with S7.39. Compound 1508215 orients similarly in the binding crevice stablishing aromatic π-π interactions between the central indole core and F183, F2.61 and F2.64, and the fluorobenzyl group with F2.57. In addition, the imidazolidinedione group H-bonds with the backbone carbonyl oxygen of V182.

In summation, from this approach, compounds 1288208, 1288239, 1508577, and 1508215 have been selected for future *in vitro* appraisal as dual CB2/TRPV1 agonists. Other compounds such as 1644371 could also be remarkable candidates for testing at TRPV1 due to its nanomolar agonist potency at CB2.

#### 3.2.3 Off-Target Evaluation

The selected hits (59824268, 1288208, 1288239, 1508577 and 1508215) have also been docked in related receptors in order to identify potential off-target effects. These molecules have been screened at CB1 and the cannabinoid-related GPCRs GPR55 and GPR18 in their active and inactive states. In addition, cannabinoid-related channels including TRPV2, TRPV3, TRPA1, and TRPM8 have also been assessed. As shown in Supplementary Table S5, by comparing docking scores to their reference orthosteric ligands we can conclude that at cannabinoid GPCRs compounds 1288208 and 1288239 might be more promiscuous showing high interaction energies at the GPR55 active and GPR18 inactive models. Moreover, compounds 1508577 and 1508215 may moderately act at CB1 whereas 1288208 and 1288239 were reported to lack binding affinity (Osman et al., 2010). 59824268 may be less selective with higher energies for the apo TRPV3 structure as well as both TRPA1 structures. 1288208 and 1288239 both show energies that are either comparable to or better than the reference ligand for each respective receptor, perhaps suggesting that the ethanolamide head group may be too promiscuous of a moiety to include when aiming to develop ligands for selective dual targeting. 1508577 shows variable activity across the TRP channels with comparable energies to the reference compounds of TRPV3 and TRPA1 in both states, with 1508215 displaying the potential for promiscuity at TRPA1.In light of these results, compounds 59824268, 1508577 and 1508215 could be prioritized as TRPV1/CB2 dual modulators. However, compounds with moderate activity at other cannabinoid targets could also be beneficial when targeting specific pathologies in which the ECS is involved or avoided when searching for more selective cannabinoid modulators.

Nonetheless, off-targets cannot be completely ruled out and not only cannabinoid-related but also other receptor families should be tested experimentally at further stages of this project.

## 4 Conclusion

Three-dimensional crystal and cryo-EM structures of GPCRs and TRP channels are being resolved at a rapid pace in the last years. The resolution of these structures are showing great impact in the field of drug discovery facilitating the emergence of successful *in silico* strategies for the identification of potential drugs targeting complex physiopathological processes.

The ECS is composed by a variety of receptors including GPCRs, TRP channels, nuclear receptors such as the PPARs (Morales et al., 2017b). Polypharmacological approaches targeting this system have already shown successful results (Fernández-Fernández et al., 2014; Malek and Starowicz, 2016; Barutta et al., 2017; Lago-Fernandez et al., 2021). For instance, a PPARγ-CB2 molecule has entered clinical trials for the treatment of systemic and multiple sclerosis (EHP-101, 2020; Palomares et al., 2018).

In this context, synergistic effects between TRPV1 and CBRs offer novel avenues for the management of pain or neurodegenerative pathologies among others. While CB1/TRPV1 dual modulators have been further studied, CB2/TRPV1 agonists have not been yet exploited. Therefore, this brief research article addresses the computational search of novel potential dual candidates for further *in vitro* and *in vivo* exploration.

Using two different virtual screening approaches we have identified hits with potential dual agonistic activity taking into account reported data and docking and druggability results. From this study, compounds 59824268, 1288208, 1288239, 1508577 and 1508215 (Supplementary Figure S5) are proposed as main candidates for future experimental appraisal. Other selected molecules reported in this article also present interesting profiles and might be worth exploring. These results provide insights into understudied scaffolds that potentially modulate CB2 and TRPV1 providing novel tools for further studies.

## Data Availability

Publicly available datasets were analyzed in this study. This data can be found here: PubChem: https://pubchem.ncbi.nlm.nih.gov/ChEMBL: https://www.ebi.ac.uk/chembl.
