# Supplementary material for: Targeting CB2 and TRPV1: Computational Approaches for the Identification of Dual Modulators
Source: Front Mol Biosci. 2022 Feb 25;9:841190. doi: 10.3389/fmolb.2022.841190 (PMC8914543; doi:10.3389/fmolb.2022.841190)
Supplement: Supplementary file 1 [file DataSheet1.docx]

Targeting CB2 and TRPV1: computational approaches for the identification of dual modulators

Paula Morales^1†*^, Chanté Muller^2†*^, Nadine Jagerovic^1^, Patricia H. Reggio^2^

^1^Medicinal Chemistry Institute, Spanish Research Council, Juan de la Cierva 3, 28006 Madrid, Spain

^2^Department of Chemistry and Biochemistry, University of North Carolina at Greensboro, Greensboro, NC 27412, USA

*** Correspondence:**Paula Morales – paula.morales@iqm.csic.es

Chanté Muller – camuller@uncg.edu

**^†^** These authors contributed equally to this work.

Supplementary Material

**Contents:**

**Table S1.** Functionality of reported cannabinoid ligands at TRPV1 and CB2.

**Figure S1.** TRPV1 sites.

**Figure S2.** Workflows for the *in silico* identification of dual TRPV1/CB2 ligands.

**Figure S3.** Docking studies of JWH133 in CB2 and TRPV1.

**Table S2.** Physicochemical descriptors calculated for potential CB2/TRPV1 modulators identified upon screening of a JWH133-related chemical library.

**Table S3.** Physicochemical descriptors calculated for potential CB2/TRPV1 modulators identified identified using the crossed-agonist strategy

**Figure S4.** Docking of AEA in S1-S4 tunnel of TRPV1.

**Table S4.** Off-target evaluation at cannabinoid-related TRPs of selected dual hits **59824268, 1288208, 1288239, 1508577** and **1508215**.

**Table S5.** Off-target evaluation at cannabinoid-related GPCRs of selected dual hits **59824268, 1288208, 1288239, 1508577** and **1508215**.

**Figure S5.** Summary of selected hits.

**Table S2.** Functionality of reported cannabinoid ligands at TRPV1 and CB2.

|  | | **TRPV1** | | | | **CB_2_** | | |
| --- | --- | --- | --- | --- | --- | --- | --- | --- |
| **Compound** | **Structure** | **Efficacy*** (%) | **Functionality**  EC_50_ (μM) | **Desensitization****  (μΜ) | **Refs.** | **Binding^$^**  K_i_ (nM) | **Functionality**  EC_50_ (nM) | **Refs.** |
| ***Endocannabinoids and endocannabinoid-like molecules*** | | | | | | | | |
| **2-AG** |  | 59.1 ± 0.3 | Agonist  0.85 ± 0.06 | 0.75 ± 0.03 | (Lowin and Straub, 2015; Petrosino et al., 2016) | 1193.9 ± 327.7 | Agonist  645.6 ± 0.4^#^ | (McPartland et al., 2007; Soethoudt et al., 2017) |
| **AEA** |  | 53.8 ± 0.2 | Agonist  0.27 ± 0.01 | 0.21 ± 0.06 | (Lowin and Straub, 2015; Petrosino et al., 2016) | 439.6 ± 95.9 | Agonist  426.6 ± 0.6^#^ | (McPartland et al., 2007; Soethoudt et al., 2017) |
| **ACPA-OH**  **(11a)** |  | 75.7 ± 2.8 | Agonist  0.047 ± 0.01 | ND | (Appendino et al., 2009) | 290 ± 6 | ND | (Appendino et al., 2009) |
| **O-1811** |  | 62.5 ± 0.8 | Agonist  0.72 ± 0.1 | ND | (Di Marzo et al., 2001) | 800.1 ± 150.2 | ND | (Di Marzo et al., 2001) |
| **Arvanil** |  | 75.4 ± 4.7 | Agonist  0.5 ± 0.2 | ND | (Di Marzo et al., 2002; Melck et al., 1999) | ND | ND | (Di Marzo et al., 2002; Melck et al., 1999) |
| **PhAR**  **derivative 12** |  | 28.9 ± 2.3 | Agonist  0.063 ± 0.004 | ND | (Appendino et al., 2006) | 22.0 ± 3.0 | Inverse Agonist^##^ | (Appendino et al., 2006) |
| ***Phytocannabinoids and phytocannabinoid-like molecules*** | | | | | | | | |
| **CBD** |  | 44.7 ± 0.02 | Agonist  1.0 ± 0.1 | 0.6 ± 0.05 | (De Petrocellis et al., 2011) | 240 (24–560) | Partial Agonist and NAM  50.1 ± 3.6^###^ | (Navarro et al., 2021; Zagzoog et al., 2020) |
| **CBDA** |  | <10 | Agonist  19.7 ± 3.9 | 89.1 ± 0.3 | (De Petrocellis et al., 2011) | 12 (4.9–77) | Partial Agonist 140 (29–310) | (Zagzoog et al., 2020) |
| **CBDV** |  | 21.4 ± 0.6 | Agonist  3.6 ± 0.7 | 10.0 ± 0.5 | (De Petrocellis et al., 2011) | 140 (96–280) | Partial Agonist 5.0 (0.46–33) | (Zagzoog et al., 2020) |
| **CBG** |  | 33.8 ± 2.3 | Agonist  1.3 ± 0.5 | 2.6 ± 0.2 | (De Petrocellis et al., 2011) | 490 (130–2500) | Partial Agonist 130 (30–550) | (Zagzoog et al., 2020) |
| **THCV** |  | 68.0 ± 1.6 | Agonist  1.5 ± 0.2 | 1.3 ± 0.1 | (De Petrocellis et al., 2011) | 47 (21–270) | Agonist  280 (49–610) | (Zagzoog et al., 2020) |
| **HU308** |  | <10 | NA | 69.0 ± 5.7 | (Soethoudt et al., 2017) | 22.7 ± 3.9 | Agonist  51.3 ± 0.3^#^ | (Hanus et al., 1999; Soethoudt et al., 2017) |
| **JWH133** |  | 24.6 ± 0.4 | Agonist  8.2 ± 0.7 | 77.7 ± 3.0 | (Soethoudt et al., 2017) | 3.4 ± 1.0 | Agonist  109.6 ± 0.4^#^ | (Huffman et al., 1999; Soethoudt et al., 2017) |
| ***Aminoalkylindole*** | | | | | | | | |
| **WIN55212-2** |  | 44.4 ± 0.9 | Agonist  19.2 ± 1.3 | 35.8 ± 2.2 | (Soethoudt et al., 2017) | 3.7 ± 0.2 | Agonist  10.9 ± 0.9^#^ | (Morales et al., 2016; Soethoudt et al., 2017) |
| *Efficacy as % of ionomycin 4 μM; ******Desensitization vs standardized agonist (0.1 μΜ capsaicin) at IC_50_ concentrations.  ^$^[^3^H]CP55,940 displacement from hCB2R cell membranes.  ^#^GTPγS assays; ^##^GTPγS assays-IC_50_ ≈ 3.2 nM (extracted from the graph); ^###^cAMP assays  NA: No activity, ND: Not determined; NAM: Negative allosteric modulator. | | | | | | | | |


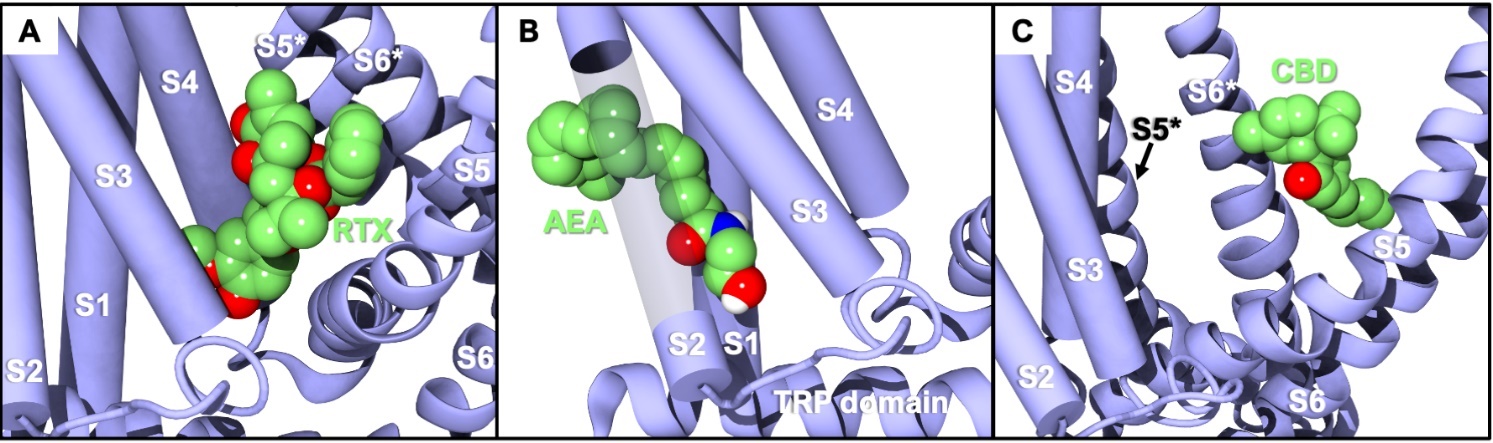


**Figure S1.** TRPV1 sites: A) RTX/TRPV1 complex in the VBP. The vanilloid binding pocket (VBP) is the location in TRPV1 in which capsaicin and resiniferatoxin (RTX) are shown to bind. RTX was used as the internal standard. The vanillyl moiety present in RTX binds deep in the VBP with the hydroxy group interacting with residues S512 and R557. Y511 interacts with one of the ester oxygens, providing stability from a key player in TRPV1 activation in response to vanilloid ligands.; B) AEA/TRPV1 complex in the S1-S4 tunnel. Previous MD results show AEA entering a novel region between the S1-S4 helices, separate from the VBP and where the putative CBD site has been reported in TRPV2.; C) CBD/TRPV1 complex between helices S5 and S6 of one monomer and S6 of another (S6*). Ligands are shown in green VDW, helices S1-S4 are shown as purple cartoon tubes and labeled, helices S5, S6, S5* and S6* are shown as cartoon ribbons and labeled. S5* and S6* are helices from the adjacent monomer. A recently published cryo-EM structure shows CBD binding in a location separate from the VBP and the tunnel between helices S5 and S6 (double check) in TRPV2. Though TRPV1 and TRPV2 share some similar features with one another, they also have a fair share of differences as analyzed in Muller 2020. The central resorcinol ring found in CBD shows two pi-stacking interactions in this putative CBD site with Y584 and F639.


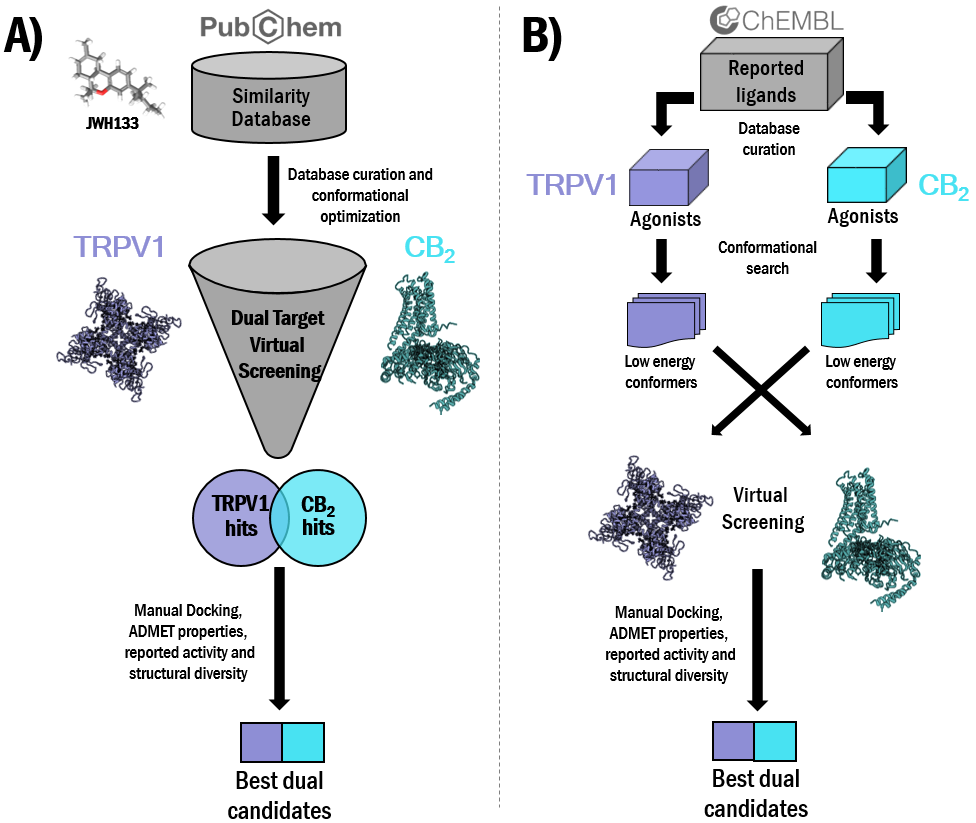


**Figure S2.** Workflows for the *in silico* identification of dual TRPV1/CB2 ligands. A) Virtual screening of JWH133 structurally related chemical databases; B) Cross-agonist HTVS.


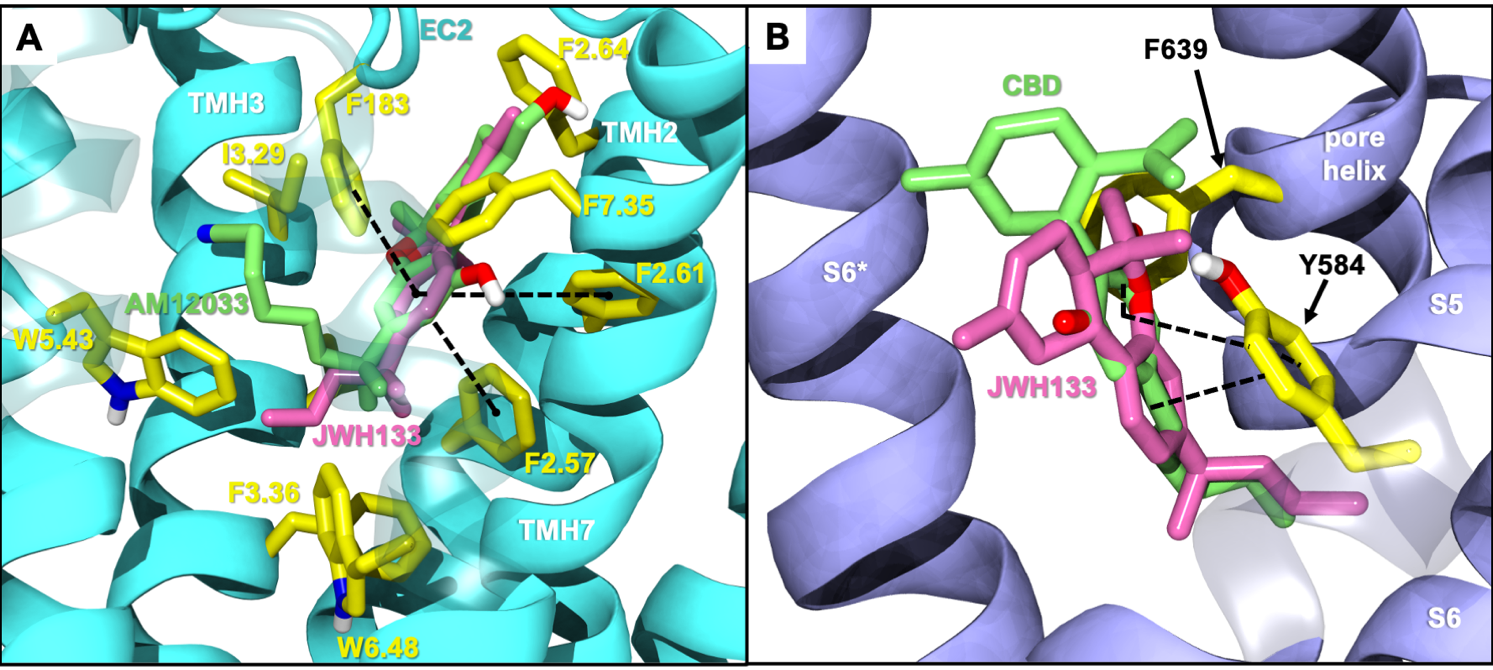


**Figure S3.** Docking studies of JWH133 in CB2 and TRPV1. A) CB2/JWH133 complex; B) TRPV1/JWH133 complex. JWH133 is displayed in magenta while reference compounds AM12033 (for CB2) and CBD (for TRPV1) are displayed in green. EC2: Extracellular loop 2; TMH: transmembrane helix.

**Table S2.** Physicochemical descriptors calculated by QikProp 3.5 integrated in Maestro (Schrödinger, LLC, New York, USA) for potential CB2/TRPV1 modulators identified upon screening of a JWH133-related chemical library.

| **Compd** | **QPlogS^a^** | **QlogBB^b^** | **QPlogHERG^c^** | **QPPCaco^d^** | **%Human oral absorption GI^e^** | **PAINS^#^** |
| --- | --- | --- | --- | --- | --- | --- |
| **JWH133** | -9.13 | 0.92 | -4.62 | 9906.04 | 100 | 0 |
| **57756957** | -8.26 | 0.52 | -5.82 | 9897.08 | 100 | 0 |
| **59824268** | -6.38 | 0.22 | -4.95 | 9902.03 | 100 | 0 |
| **123533625** | -9.12 | 0.39 | -5.98 | 9734.16 | 100 | 1 |
| **153641693** | -8.79 | 0.38 | -5.76 | 9938.04 | 100 | 0 |
| **151332252** | -8.54 | 0.25 | -6.45 | 8901.59 | 100 | 0 |
| **^a^**Predicted aqueous solubility [-6.5/0.5]; ^b^Predicted log of the brain/blood partition coefficient [-3.0/1.2]; ^c^HERG K^+^ Channel Blockage (log IC_50_) [concern below -5]; ^d^Apparent Caco-2 cell permeability in nm/s [<25 poor, >500 excellent]; ^e^Human Oral Absorption in GI [<25% is poor]. [range of 95% of drugs]. **^#^**Number of structural alerts as calculated using the swissADME webserver (Daina et al., 2017). | | | | | | |

**Table S3.** Physicochemical descriptors calculated by QikProp 3.5 integrated in Maestro (Schrödinger, LLC, New York, USA) for potential CB2/TRPV1 modulators identified using the crossed-agonist strategy.

| **Compd** | **QPlogS^a^** | **QlogBB^b^** | **QPlogHERG^c^** | **QPPCaco^d^** | **%Human oral absorption GI^e^** | **PAINS^#^** |
| --- | --- | --- | --- | --- | --- | --- |
| **AM12033** | -7.38 | -1.62 | -4.95 | 353.08 | 100 | 0 |
| **1508577** | -4.84 | -1.56 | -5.91 | 264.91 | 84 | 0 |
| **1508215** | -4.86 | -0.99 | -5.07 | 376.21 | 93 | 0 |
| **1574712** | -5.32 | -0.99 | -5.47 | 446.52 | 100 | 0 |
| **1383349** | -5.49 | -0.40 | -7.00 | 2405.32 | 100 | 0 |
| **1347563** | -5.46 | -0.14 | -6.73 | 3176.79 | 100 | 0 |
| **TRPV1** | | | | | |  |
| **Compd** | **QPlogS^a^** | **QlogBB^b^** | **QPlogHERG^c^** | **QPPCaco^d^** | **%Human oral absorption GI^e^** | **PAINS^#^** |
| **AEA** | -6.02 | -1.52 | -4.69 | 994.90 | 100 | 0 |
| **1288208*** | -3.04 | -1.33 | -4.58 | 395.41 | 86 | 0 |
| **1288239** | -3.65 | -1.51 | -5.16 | 287.31 | 86 | 0 |
| **CBD** | -5.92 | -0.47 | -4.87 | 2519.02 | 100 | 0 |
| **1644371** | -5.42 | 0.15 | -5.59 | 2986.65 | 100 | 0 |
| **3114522** | -8.35 | -1.19 | -7.69 | 774.81 | 100 | 0 |
| **RTX** | -8.81 | -1.97 | -7.17 | 289.99 | 81 | 1 |
| **3353818** | -4.77 | 1.38 | -6.10 | 7.99 | 51 | 0 |
| **1288208*** | -3.04 | -1.33 | -4.58 | 395.41 | 86 | 0 |
| **1644673** | -6.13 | 0.67 | -8.15 | 1152.10 | 100 | 0 |
| **^a^**Predicted aqueous solubility [-6.5/0.5]; ^b^Predicted log of the brain/blood partition coefficient [-3.0/1.2]; ^c^HERG K^+^ Channel Blockage (log IC_50_) [concern below -5]; ^d^Apparent Caco-2 cell permeability in nm/s [<25 poor, >500 excellent]; ^e^Human Oral Absorption in GI [<25% is poor]. [range of 95% of drugs]; *Compound selected for both tunnel and VBP docking. **^#^**Number of structural alerts as calculated using the swissADME webserver (Daina et al., 2017). | | | | | | |


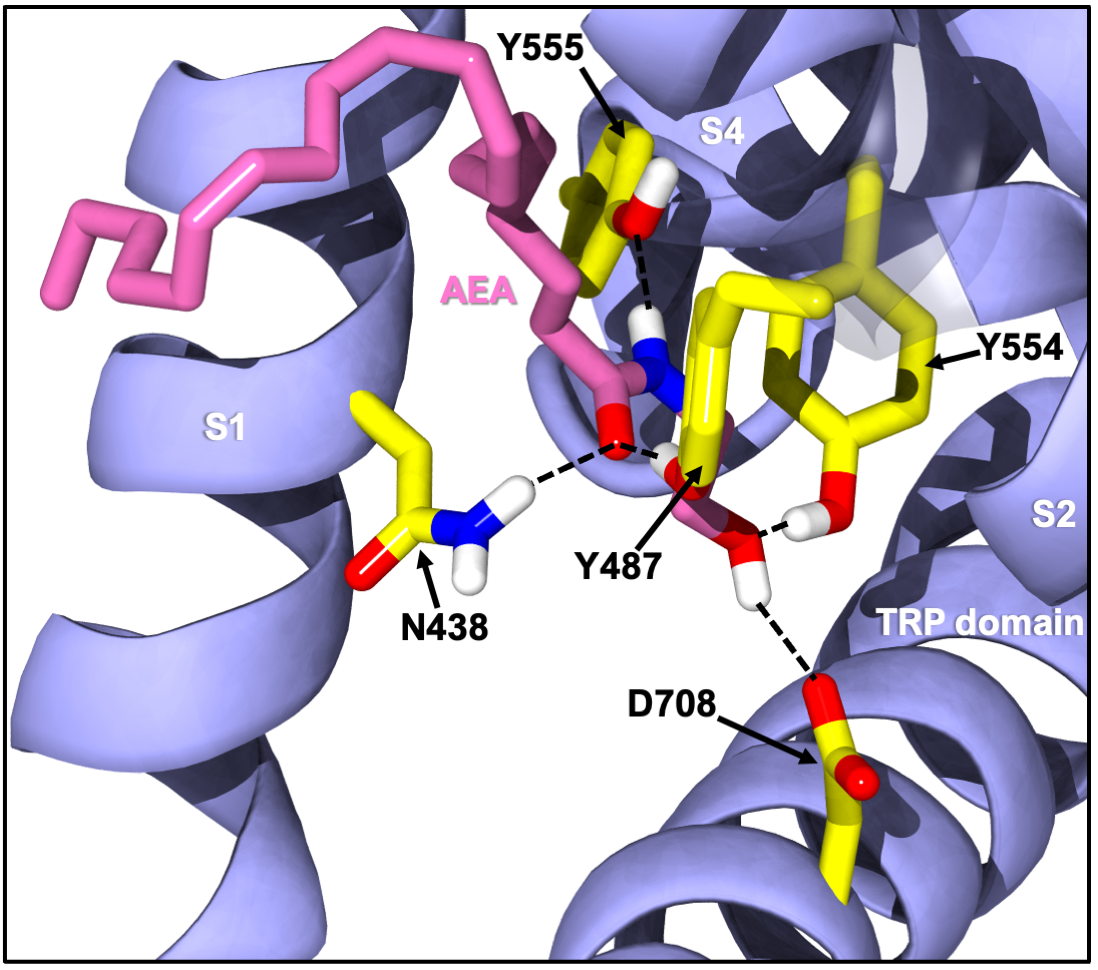


Figure S4. The docking output of AEA (pink) in the S1-S4 tunnel region of TRPV1. Headgroup interactions consist of H-bonds with N438, D708, Y487, Y554, and Y555 (all yellow).

**Table S4.** Off-target evaluation at cannabinoid-related TRPs of selected dual hits 59824268, 1288208, 1288239, 1508577 and 1508215. The following compounds have been included as reference for each target: TRPV2 agonist (CBD), TRPV3 agonist (2-ABP), TRPA1 agonist (benzothioformamide), and TRPM8 agonist (WS-12).

| **Compd** | **Structure** | **TRPV2 active^a^** | **TRPV2 apo^ah^** | **TRPV3**  **active^b^** | **TRPV3**  **apo^ch^** | **TRPA1 active^dh^** | **TRPA1 apo^ehi^** | **TRPM8 active^f,h^** | **TRPM8 apo^gh^** | |
| --- | --- | --- | --- | --- | --- | --- | --- | --- | --- | --- |
| **CBD^a^** |  | -10.79 | -8.71 | - | - | - | - | - | - | |
| **2-ABP^c^** |  | - | - | -6.99 | -4.08 | - | - | - | - | |
| **Benzothioformamide^d^** |  | - | - | - | - | -7.98 | -5.21 | - | - | |
| **WS-12^f^** |  | - | - | - | - | - | - | -9.43 | -4.56 | |
| **59824268** |  | -8.24 | -7.60 | -6.11 | -5.11 | -6.28 | -6.48 | -7.07 | -4.53 | |
| **1288208** |  | -10.28  VBP | -9.13  VBP | -7.18  TNL | -6.42  TNL | -7.04  VBP | -7.60  VBP | -8.35  VBP | -6.96  VBP | |
| **1288239** |  | -9.83  VBP | -8.98  TNL | -8.76  TNL | -7.35  TNL | -7.98  VBP | -7.43  VBP | -9.43  VBP | -6.58  VBP | |
| **1508577** |  | -5.58 | -6.50 | -6.40 | -3.92 | -4.13 | -4.56 | -4.80 | -3.87 | |
| **1508215** |  | -5.24 | -8.77 | -5.47 | -5.94 | -6.21 | -6.88 | -6.93 | -2.90 | |
| ^a^(Pumroy et al., 2019); ^b^ (Zubcevic et al., 2018); ^c^(Singh et al., 2018); ^d^(Suo et al., 2020); ^e^(Zhao et al., 2020); ^f^(Yin et al., 2019); ^g^(Diver et al., 2019); ^h^Reference ligands were tested at each apo structure to determine their docking score at the inactive structure. TRPV2, TRPV3, and TRPM8 showed decreases in the scoring. ^i^The apo state in TRPA1 indicates a higher score than the active structure. Since benzothioformamide covalently binds intracellularly, the resolved lipid found in the transmembrane region was used as the inactive reference. Since the ligands tested are more lipophilic than the agonist used, this site was used to dock the selected ligands. ^*^These ligands were screened in the VBP and the tunnel of each channel due to their docking withing the tunnel of TRPV1. The best score of the two recorded and indicated here as “VBP” or “TNL”. | | | | | | | | | |  |

**Table S5.** Off-target evaluation at cannabinoid-related GPCRs of selected dual hits 59824268, 1288208, 1288239, 1508577 and 1508215. The following compounds have been included as reference for each target: CB1 agonist (AM841), CB1 antagonist (Taranabant), GPR55 agonist (ML184), GPR55 antagonist (ML193), GPR18 agonist (S5), and GPR18 antagonist (PBS-CB5).

| **Compd** | **Structure** | **CB1 active^a^** | **CB1 inactive^b^** | **GPR55**  **active^c^** | **GPR55**  **Inactive^d^** | **GPR18**  **active^e^** | **GPR18**  **Inactive^e^** |
| --- | --- | --- | --- | --- | --- | --- | --- |
| **AM841^a^** |  | -12.07 | - | - | - | - | - |
| **Taranabant^b^** |  | - | -14.89 | - | - | - | - |
| **ML184^c^** |  | - | - | -10.31 | - | - | - |
| **ML193^d^** |  | - | - | - | -6.98 | - | - |
| **S5^f^** |  | - | - | - | - | -7.19 | - |
| **PSB-CB5^g^** |  | - | - | - | - | - | -6.79 |
| **59824268** |  | -7.19 | -8.46 | -7.85 | -4.37 | -4.40 | -4.15 |
| **1288208** |  | Inactive^h^ | Inactive^h^ | -10.51 | -5.75 | -3.20 | -6.03 |
| **1288239** |  | Inactive^h^ | Inactive^h^ | -10.16 | -4.31 | -5.56 | -5.91 |
| **1508577** |  | -7.85 | -9.27 | -8.03 | -1.65 | -2.40 | -3.50 |
| **1508215** |  | -8.03 | -9.51 | -7.49 | -5.97 | -3.18 | -4.64 |
| ^a^PDB-ID: 6KPG (Hua et al., 2020);  ^b^PDB-ID: 5U09 (Shao et al., 2016); ^c^(Lingerfelt et al., 2017); ^d^(Kotsikorou et al., 2013); ^e^(Sotudeh et al., 2019); ^f^(Jagerovic et al., 2020);  ^g^(Rempel et al., 2014); ^h^Reported binding assays indicate that these compounds do not show activity at CB1[K_i_ > 10 µM (Osman et al., 2010)] | | | | | | | |

**Figure S5.** Summary of selected hits.


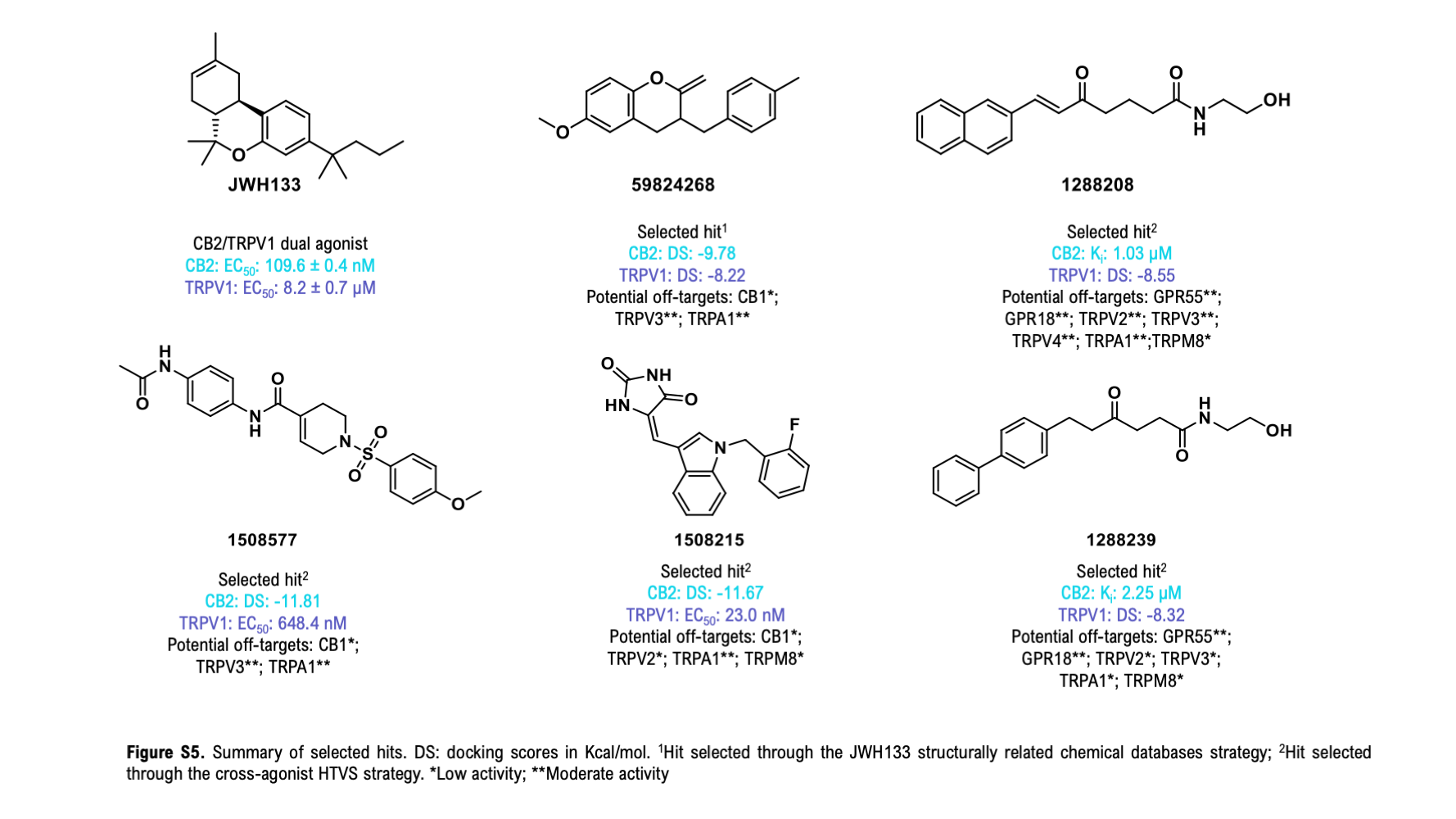


**Figure S5.** Summary of selected hits. DS: docking scores in Kcal/mol. ^1^Hit selected through the JWH133 structurally related chemical databases strategy; ^2^Hit selected through the cross-agonist HTVS strategy. *Low activity; **Moderate activity.

**REFERENCES**

Appendino, G., Cascio, M. G., Bacchiega, S., Moriello, A. S., Minassi, A., Thomas, A., et al. (2006). First “hybrid” ligands of vanilloid TRPV1 and cannabinoid CB 2 receptors and non-polyunsaturated fatty acid-derived CB 2-selective ligands. *FEBS Lett.* 580, 568–574. doi:10.1016/j.febslet.2005.12.069.

Appendino, G., Ligresti, A., Minassi, A., Cascio, M. G., Allarà, M., Taglialatela-Scafati, O., et al. (2009). Conformationally constrained fatty acid ethanolamides as cannabinoid and vanilloid receptor probes. *J. Med. Chem.* 52, 3001–3009. doi:10.1021/jm900130m.

Daina, A., O, M., and Zoete, V. (2017). SwissADME: a free web tool to evaluate pharmacokinetics, drug-likeness and medicinal chemistry friendliness of small molecules. *Sci. Rep.* 7, 42717. doi:10.1038/srep42717.

De Petrocellis, L., Ligresti, A., Moriello, A. S., Allar??, M., Bisogno, T., Petrosino, S., et al. (2011). Effects of cannabinoids and cannabinoid-enriched Cannabis extracts on TRP channels and endocannabinoid metabolic enzymes. *Br. J. Pharmacol.* 163, 1479–1494. doi:10.1111/bph.2011.163.issue-7.

Di Marzo, V., Bisogno, T., De Petrocellis, L., Brandi, I., Jefferson, R. G., Winckler, R. L., et al. (2001). Highly selective CB1cannabinoid receptor ligands and novel CB1/VR1vanilloid receptor ୀhybridୁ ligands. *Biochem. Biophys. Res. Commun.* 281, 444–451. doi:10.1006/bbrc.2001.4354.

Di Marzo, V., Griffin, G., De Petrocellis, L., Brandi, I., Bisogno, T., Williams, W., et al. (2002). A structure/activity relationship study on arvanil, an endocannabinoid and vanilloid hybrid. *J. Pharmacol. Exp. Ther.* 300, 984–991. doi:10.1124/jpet.300.3.984.

Diver, M. M., Cheng, Y., and Julius, D. (2019). Structural insights into TRPM8 inhibition and desensitization. *Science (80-. ).* 365, 1434–1440. doi:10.1126/science.aax6672.

Gao, Y., Cao, E., Julius, D., and Cheng, Y. (2016). TRPV1 structures in nanodiscs reveal mechanisms of ligand and lipid action. *Nature* 534, 347–51. doi:10.1038/nature17964.

Hanus, L., Breuer, A., Tchilibon, S., Shiloah, S., Goldenberg, D., Horowitz, M., et al. (1999). HU-308: a specific agonist for CB(2), a peripheral cannabinoid receptor. *Proc. Natl. Acad. Sci. U. S. A.* 96, 14228–33. Available at: http://www.pubmedcentral.nih.gov/articlerender.fcgi?artid=24419&tool=pmcentrez&rendertype=abstract [Accessed February 5, 2015].

Huffman, J. W., Liddle, J., Yu, S., Aung, M. M., Abood, M. E., Wiley, J. L., et al. (1999). 3-(1’,1’-Dimethylbutyl)-1-deoxy-Δ8-THC and related compounds: Synthesis of selective ligands for the CB2 receptor. *Bioorganic Med. Chem.* 7, 2905–2914. doi:10.1016/S0968-0896(99)00219-9.

Lowin, T., and Straub, R. H. (2015). Cannabinoid-based drugs targeting CB1 and TRPV1, the sympathetic nervous system, and arthritis. *Arthritis Res. Ther.* 17, 226. doi:10.1186/s13075-015-0743-x.

McPartland, J. M., Glass, M., and Pertwee, R. G. (2007). Meta-analysis of cannabinoid ligand binding affinity and receptor distribution: interspecies differences. *Br. J. Pharmacol.* 152, 583–593. doi:10.1038/sj.bjp.0707399.

Melck, D., Bisogno, T., De Petrocellis, L., Chuang, H. H., Julius, D., Bifulco, M., et al. (1999). Unsaturated long-chain N-acyl-vanillyl-amides (N-AVAMs): Vanilloid receptor ligands that inhibit anandamide-facilitated transport and bind to CB1 cannabinoid receptors. *Biochem. Biophys. Res. Commun.* 262, 275–284. doi:10.1006/bbrc.1999.1105.

Morales, P., Gómez-Cañas, M., Navarro, G., Hurst, D. P., Carrillo-Salinas, F. J., Lagartera, L., et al. (2016). Chromenopyrazole, a Versatile Cannabinoid Scaffold with in Vivo Activity in a Model of Multiple Sclerosis. *J. Med. Chem.* 59, 6753–6771. doi:10.1021/acs.jmedchem.6b00397.

Navarro, G., Gonzalez, A., Sánchez-Morales, A., Casajuana-Martin, N., Gómez-Ventura, M., Cordomí, A., et al. (2021). Design of Negative and Positive Allosteric Modulators of the Cannabinoid CB 2 Receptor Derived from the Natural Product Cannabidiol. *J. Med. Chem.* 64, 9354–9364. doi:10.1021/acs.jmedchem.1c00561.

Petrosino, S., Schiano Moriello, A., Cerrato, S., Fusco, M., Puigdemont, A., De Petrocellis, L., et al. (2016). The anti-inflammatory mediator palmitoylethanolamide enhances the levels of 2-arachidonoyl-glycerol and potentiates its actions at TRPV1 cation channels. *Br. J. Pharmacol.* 173, 1154–1162. doi:10.1111/bph.13084.

Pumroy, R. A., Samanta, A., Liu, Y., Hughes, T., Zhao, S., Yudin, Y., et al. (2019). Molecular mechanism of TRPV2 channel modulation by cannabidiol. doi:10.7554/eLife.48792.001.

Singh, A. K., McGoldrick, L. L., and Sobolevsky, A. I. (2018). Structure and gating mechanism of the transient receptor potential channel TRPV3. *Nat. Struct. Mol. Biol.* 25, 805–813. doi:10.1038/s41594-018-0108-7.

Soethoudt, M., Grether, U., Fingerle, J., Grim, T. W., Fezza, F., de Petrocellis, L., et al. (2017). Cannabinoid CB2 receptor ligand profiling reveals biased signalling and off-target activity. *Nat. Commun.* 8, 13958. doi:10.1038/ncomms13958.

Suo, Y., Wang, Z., Zubcevic, L., Hsu, A. L., He, Q., Borgnia, M. J., et al. (2020). Structural Insights into Electrophile Irritant Sensing by the Human TRPA1 Channel. *Neuron* 105, 882-894.e5. doi:10.1016/j.neuron.2019.11.023.

Yin, Y., Le, S. C., Hsu, A. L., Borgnia, M. J., Yang, H., and Lee, S. Y. (2019). Structural basis of cooling agent and lipid sensing by the cold-activated TRPM8 channel. *Science (80-. ).* 363. doi:10.1126/science.aav9334.

Zagzoog, A., Mohamed, K. A., Kim, H. J. J., Kim, E. D., Frank, C. S., Black, T., et al. (2020). In vitro and in vivo pharmacological activity of minor cannabinoids isolated from Cannabis sativa. *Sci. Rep.* 10, 1–13. doi:10.1038/s41598-020-77175-y.

Zhao, J., Lin King, J. V., Paulsen, C. E., Cheng, Y., and Julius, D. (2020). Irritant-evoked activation and calcium modulation of the TRPA1 receptor. *Nature* 585, 141–145. doi:10.1038/s41586-020-2480-9.

Zubcevic, L., Herzik, M. A., Wu, M., Borschel, W. F., Hirschi, M., Song, A. S., et al. (2018). Conformational ensemble of the human TRPV3 ion channel. *Nat. Commun.* 9. doi:10.1038/s41467-018-07117-w.
